# FABP3, FABP4, and heart rate variability among patients with chronic schizophrenia

**DOI:** 10.3389/fendo.2023.1165621

**Published:** 2023-05-15

**Authors:** Wei-Chin Hung, Teng-Hung Yu, Cheng-Ching Wu, Thung-Lip Lee, I-Ting Tsai, Chin-Feng Hsuan, Chun-Yu Chen, Fu-Mei Chung, Yau-Jiunn Lee, Wei-Hua Tang

**Affiliations:** ^1^ Division of Cardiology, Department of Internal Medicine, E-Da Hospital, I-Shou University, Kaohsiung, Taiwan; ^2^ School of Medicine, College of Medicine, I-Shou University, Kaohsiung, Taiwan; ^3^ Division of Cardiology, Department of Internal Medicine, E-Da Cancer Hospital, I-Shou University, Kaohsiung, Taiwan; ^4^ School of Medicine for International Students, College of Medicine, I-Shou University, Kaohsiung, Taiwan; ^5^ Department of Emergency, E-Da Hospital, I-Shou University, Kaohsiung, Taiwan; ^6^ Division of Cardiology, Department of Internal Medicine, E-Da Dachang Hospital, I-Shou University, Kaohsiung, Taiwan; ^7^ Division of General Neurology, Neurological Institute, Taipei Veterans General Hospital, Taipei, Taiwan; ^8^ Department Head, Lee’s Endocrinologic Clinic, Pingtung, Taiwan; ^9^ Division of Cardiology, Department of Internal Medicine, Taipei Veterans General Hospital, Yuli Branch, Hualien, Taiwan; ^10^ Faculty of Medicine, School of Medicine, National Yang Ming Chiao Tung University, Taipei, Taiwan

**Keywords:** schizophrenia, cardiovascular disease, heart rate variability, high frequency, low-frequency, fatty acid-binding protein

## Abstract

**Introduction:**

The prevalence of cardiovascular disease (CVD) and CVD-related deaths in patients with schizophrenia is high. An elevated risk of CVD has been associated with low heart rate variability (HRV). There is increasing evidence that fatty acid-binding protein (FABP)3 and FABP4 play roles in the development and progression of CVD. This study aimed to explore the association of circulating FABP3/FABP4 levels with HRV in patients with chronic schizophrenia.

**Methods:**

We included 265 consecutive patients with chronic schizophrenia who attended a disease management program. We used an enzyme-linked immunosorbent assay for the measurement of plasma concentrations of FABP3 and FABP4. Standard HRV was recorded at baseline following a standard protocol. Mean high- and low-frequency (HF/LF) HRV values were analyzed by tertile of FABP3 and FABP4 using one-way analysis of variance, and linear regression analysis was performed to assess trends.

**Results:**

A positive association between FABP3 and creatinine was found in multiple regression analysis. In addition, negative associations between levels of hematocrit, hemoglobin, HF HRV, and estimated glomerular filtration rate (eGFR) with FABP3 were also found. Moreover, positive associations between FABP4 with body mass index, diabetes mellitus, hypertension, systolic blood pressure, low-density lipoprotein-cholesterol, triglycerides, creatinine, and FABP3 were found. Furthermore, negative associations between levels of high-density lipoprotein-cholesterol, eGFR, and HF HRV with FABP4 were found. We also found a significant inverse association between FABP3 and HF HRV (p for trend = 0.008), and significant inverse associations between FABP4 with HF and LF HRV (p for trend = 0.007 and 0.017, respectively).

**Discussion:**

Together, this suggests that elevated levels of FABP3 and FABP4 may be linked to health problems related to CVD in patients with chronic schizophrenia.

## Introduction

The cardiac biomarker fatty acid-binding protein 3 (FABP3) has been used as a diagnostic tool for acute myocardial infarction (AMI) and non-ST-elevation myocardial infarction (1). It is low-molecular weight (15 kDa) cytosolic protein specific to cardiomyocytes, and it represents 5 to 15% of all cardiomyocyte-related cytosolic proteins (2). In the mitochondria, FABP3 has been shown to be involved in the intracellular transport of fatty acids for β-oxidation (3). Moreover, associations between FABP3 and other clinical parameters have also been reported in AMI patients, including high-sensitive troponin T, N-terminal pro-brain natriuretic peptide, ejection fraction, C-reactive protein (CRP), leucocytes, and length of hospital stay (4). FABP3 has been reported as a useful marker of both myocardial damage and also to induce inflammation along with vascular smooth muscle cell growth and migration (5). FABP4 is mostly expressed in macrophages and adipocytes, and it has been strongly associated with the development of insulin resistance and atherosclerosis in association with low-grade inflammation (6). FABP4 may act as an adipokine, and its release from adipocytes has been shown to be through a non-classical pathway related to lipolysis (7), which is mainly activated by catecholamines during activation of sympathetic nervous system. An increase in circulating FABP4 levels has been associated with hypertension, diabetes mellitus, insulin resistance, obesity, atherosclerosis, cardiovascular events and cardiac dysfunction (6, 8).

Heart rate variability (HRV) is defined as fluctuations in the time between heartbeats. HRV has been used to assess autonomic function, with low HRV indicating impaired autonomic function (9). Therefore, HRV can be a useful measure of cardiovascular health and stress (10). HRV decreases with age (11) and it has been associated with cognitive decline (12), while an increase in HRV has been associated with longevity and healthy aging (13). Consequently, HRV can be used to assess both physiological and neurological status. Patients with schizophrenia have been shown to have low HRV (14), and a low HRV has also been associated with a worse severity of symptoms and cognitive test scores, diabetes mellitus (15), ongoing subclinical inflammation (16), and higher risks of cardiovascular disease (CVD)-related morbidity and mortality (17, 18), and poorer quality of life (19, 20). Hence, investigating risk factors associated with reduced HRV in patients with schizophrenia is important.

Increasing evidence suggests that FABP3 and FABP4 may play roles in the progression and development of CVD (5, 6, 8). Furthermore, our previous studies found that plasma FABP3 could be used as a surrogate for reduced ejection fraction and abnormal corrected QT (QTc) interval, and that in patients with stable angina (21), plasma FABP4 level may also be associated with an abnormal QTc interval (22). However, few studies have investigated the associations of FABP3 and FABP4 levels with HRV. Therefore, the aim of this study was to examine whether FABP3 and FABP4 were associated with HRV in a cohort of patients with chronic schizophrenia.

## Materials and methods

### Participants

This cross-sectional study included 265 patients with chronic schizophrenia with stable status from inpatient wards at Taipei Veterans General Hospital, Yuli Branch between December 02, 2021 to December 01, 2022. The Diagnostic and Statistical Manual of Mental Disorders IV was used to confirm the diagnosis of schizophrenia. We excluded those with a major affective disorder (including mania, major depression, bipolar disorder and schizoaffective disorder), acute physical illnesses and fever. In addition, we excluded those who received steroid, antipyretic, antibiotic, or anti-inflammatory agent therapy. Before enrollment, associations among plasma levels of FABP3, FABP4, and CRP with sociodemographic characteristics were evaluated. All of the enrolled patients provided written informed consent. The Human Research Ethics Committee of Kaohsiung E-Da Hospital approved this study which was conducted following the Declaration of Helsinki.

Sociodemographic characteristics including sex, age, mental status, smoking, drinking, betel quid chewing, weight, height, and body mass index (BMI, kg/m^2^) were recorded. Each patient underwent neurological and physical examinations.

### Laboratory measurements

Blood samples were collected after fasting for a minimum of 8 hours. Serum uric acid and complete blood count were measured using standard methods with an automatic biochemical analysis system (Hitachi 7170A, Tokyo, Japan) as reported previously (23). In addition, serum creatinine was measured using the Jaffe method. The same system was used for lipid measurements, [including plasma triglycerides, total cholesterol, high-density lipoprotein cholesterol (HDL-C), and low-density lipoprotein cholesterol (LDL-C)], as well as glucose and glycated hemoglobin (HbA1c). Peripheral leukocyte count was measured using an automated cell counter (XE-2100 Hematology Alpha Transportation System, Sysmex Corporation, Kobe, Japan). Renal function (estimated glomerular filtration rate, eGFR) was calculated using the CKD-EPI two-concentration race equation as reported previously (24). Enzyme-linked immunosorbent assay kits were used to measure the concentrations of plasma FABP3 and FABP4 (Invitrogen, Thermo Fisher Scientific Inc., USA and R&D Systems Inc., Minneapolis, MN, USA), with intra- and inter-assay coefficients of variation of 3.4% to 5.8% (n = 3) and 3.1% to 6.2% (n = 4), respectively, for FABP4, and 3.9% (n = 8) and 6.2% (n = 8), respectively, for FABP3. Plasma levels of high-sensitivity CRP were measured using a chemistry analyzer (IMMAGE, Beckman Coulter, Brea, CA, USA); the intra-assay coefficient of variation ranged from 4.2% to 8.7%, and the detection limit was 0.2 mg/L. All measurements were performed in duplicate in a single experiment.

### Definitions

Patients with hypertension were classified as those with systolic/diastolic blood pressures (SBP/DBP) ≥140/90 mmHg, or prescriptions for antihypertension therapy. Patients with diabetes mellitus were classified as those with HbA1c ≥6.5% (48 mmol/mol), a fasting blood glucose level ≥126 mg/dL (7.0 mmol/L), 2-hour blood glucose ≥200 mg/dL (11.1 mmol/L) according to the American Diabetes Association 2016 Guidelines (25), or receiving antidiabetic therapy. Hyperlipidemia was defined as triglycerides >180 mg/dl, and/or LDL-C >130 mg/dl, and/or total cholesterol >200 mg/dl, and/or HDL-C level <40 mg/dl, or receiving lipid disorder treatment.

### HRV assessment

The 5-minute HRV are reported according to the Reporting Articles on Psychiatry and HRV Guidelines and the procedure was based on the standard method and has been described previously (26–29). An HRV analyzer (SS101; Enjoy Research Inc., Hualien, Taiwan) was used for acquisition, storage, and processing of ECG signals. All patients are in a lying (supine) position during examination and are breathing spontaneously during ECG recording. In brief, all the signals were recorded by an 8-bit analog-to-digital converter with a sampling rate of 512 Hz. The digitized ECG signals were analyzed and stored immediately for verification. The computer algorithm identified each QRS complex and rejected each abnormal signal (such as ventricular premature complex or noise). Stationary R-R interval values were resampled and interpolated at a rate of 7.11 Hz for producing continuity in the time domain. This interpolation produced 2048 data points over 288 seconds. Through Fourier transformation, the power spectrum was then quantified into the frequency-domain measurements, which were defined in previous studies (27, 28). The frequency domain indexes included total power (TP), very low-frequency power (VLF; 0.003-0.04 Hz), low-frequency power (LF; 0.04-0.15 Hz), high-frequency power (HF; 0.15-0.40 Hz), LF/HF, and normalized LF (LF%). LF% was defined as LF/(TP − VLF) × 100. For correcting the skewed distributions, TP, VLF, LF, HF, and LF/HF were logarithmically transformed. HF is a parasympathetic index, whereas LF represent sympathetic function. Here, we focused on distributions of major heart rate oscillations in HF and LF bands according to previous studies (30–32).

### Statistical analysis

Data normality was evaluated using the Kolmogorov-Smirnov test. Normally distributed continuous variables are shown as mean ± SD and compared using the unpaired Student’s *t*-test, and non-normally distributed continuous variables are shown as median (interquartile range). Categorical variables are shown as number (%), and compared using the chi-square test. As high-sensitivity CRP, FABP3, FABP4, and HRV had skewed distribution, logarithmically transformed values were used in the analysis. Associations were examined between plasma FABP4 and FABP3 and other variables using simple and multiple linear regression analyses. Mean HRV values for FABP3 and FABP4 tertiles were obtained using one-way analysis of variance for heterogeneity. P-values for trends were obtained from linear regression analysis. A p-value <0.05 was considered statistically significant. The analyses were performed with JMP version 7.0 for Windows (SAS Institute, Cary, NC, USA).

## Results

### Characteristics of the patients by sex

The mean (± SD) age of the patients was 55.5 ± 11.0 years (range 26-83 years), 155 were male (58.5%) and 110 were female (41.5%) (**Table 1**). the female patients had a longer duration of schizophrenia; higher levels of hyperlipidemia, total cholesterol, HDL-C, eGFR, and FABP3, and lower levels of uric acid, creatinine, albumin, hematocrit, hemoglobin, and high-sensitivity CRP, white blood cell count and were older than the male patients. However, HF HRV and LF HRV did not differ between female patients and male patients (**Table 1**). Furthermore, when we have calculated the HF HRV and LF HRV by age groups (patients >50 years and patients ≤50 years), we found that HF HRV and LF HRV did not differ between patients with >50 years and ≤50 years (3.9 ± 1.8 ms^2^ *vs*. 3.9 ± 2.2 ms^2^, p=0.963 for HF HRV, and 4.1 ± 1.7 ms^2^ *vs*. 4.2 ± 2.0 ms^2^, p=0.604 for LF HRV). Moreover, when we have calculated the HF HRV and LF HRV by with and without antihypertensive drugs, we found that HF HRV and LF HRV did not differ between patients with and without antihypertensive drugs (3.7 ± 1.7 ms^2^ *vs*. 4.1 ± 2.1 ms^2^, p=0.127 for HF HRV, and 4.0 ± 1.6 ms^2^ *vs*. 4.3 ± 1.9 ms^2^, p=0.116 for LF HRV). In addition, in the present study, there were 83 (31.3%) and 182 (68.7%) patients received typical antipsychotics and atypical antipsychotics. When calculated the HF HRV and LF HRV by antipsychotic treatment (typical antipsychotics and atypical antipsychotics), we found that HF HRV and LF HRV did not differ between patients received typical antipsychotics and atypical antipsychotics (3.7 ± 2.2 ms^2^ *vs*. 4.0 ± 1.8 ms^2^, p=0.348 for HF HRV, and 3.9 ± 2.0 ms^2^ *vs*. 4.3 ± 1.6 ms^2^, p=0.116 for LF HRV) (data not shown). These results may exclude the confounding effect of age, sex, drug of antihypertensive, and antipsychotic treatment on the HRV data of present study.

**Table 1:** Demographics and other characteristics of the study population by gender.

Characteristics	All	Women	Men	p-value
Number	265	110	155	
Age (years)	55.5±11.0	59.1±11.0	52.9±10.2	<0.0001
Duration of schizophrenia (years)	30.4±11.0	33.8±11.6	28.1±10.0	<0.0001
Diabetes mellitus (n, %)	56 (21.1)	28 (25.5)	28 (18.1)	0.147
Hypertension (n, %)	124 (46.8)	58 (52.7)	66 (42.6)	0.103
Hyperlipidemia (n, %)	42 (15.9)	24 (21.8)	18 (11.6)	0.025
Body mass index (kg/m^2^)	24.2±4.5	23.7±5.0	24.5±4.1	0.140
Systolic blood pressure (mmHg)	123±14	122±15	123±13	0.581
Diastolic blood pressure (mmHg)	75±11	75±11	75±12	0.666
Fasting glucose (mg/dL)	95.0±24.4	95.5±27.0	94.6±22.3	0.773
HbA1c (%)	5.8±0.7	5.8±0.7	5.9±0.7	0.412
Total cholesterol (mg/dL)	154.4±32.7	162.8±31.9	148.2±31.9	0.0004
Triglyceride (mg/dl)	97.7±46.9	99.4±46.3	96.4±47.5	0.606
HDL cholesterol (mg/dl)	53.4±15.5	60.2±15.5	48.4±13.5	<0.0001
LDL cholesterol (mg/dl)	89.9±29.2	92.2±27.9	87.1±30.6	0.218
Uric acid (mg/dl)	4.7±2.0	3.9±1.5	5.2±2.1	0.001
Creatinine (mg/dl)	0.85±0.44	0.75±0.29	0.92±0.51	0.002
eGFR (ml/min/1.73m^2^)	116.5±37.6	131.1±41.0	106.2±31.2	<0.0001
Albumin (g/dl)	3.9±0.5	3.7±0.5	4.0±0.5	0.021
Hematocrit (%)	36.6±4.8	34.6±3.9	38.0±4.8	<0.0001
Hemoglobin (g/dl)	12.6±1.7	11.8±1.4	13.2±1.6	<0.0001
Hs-CRP (mg/L)^†^	0.15 (0.06-0.32)	0.12 (0.04-0.22)	0.17 (0.06-0.51)	0.003
White blood cell count (×10^9^/L)	6516±2855	5996±2264	6886±3167	0.013
Fatty acid-binding protein 3 (ng/mL)^†^	1.08 (0.88-1.49)	1.11 (0.94-1.74)	1.07 (0.85-1.36)	0.024
Fatty acid-binding protein 4 (ng/mL)^†^	15.3 (10.6-23.0)	17.2 (11.9-25.8)	14.6 (9.4-21.5)	0.067
HF HRV (ms^2^)^†^	4.0 (2.6-5.2)	4.0 (2.7-4.9)	4.0 (2.6-5.3)	0.630
LF HRV (ms^2^)^†^	4.2 (3.0-5.3)	4.0 (2.9-5.1)	4.4 (3.5-5.3)	0.320

Data are presented as mean ± SD or median (interquartile range). HDL, high-density lipoprotein cholesterol; LDL, low-density lipoprotein cholesterol; eGFR, estimated glomerular filtration rate; Hs-CRP, high-sensitivity C-reactive protein; HF, absolute power of the high-frequency band; LF, absolute power of the low frequency band; HRV, heart rate variability.

^†^Variable was first log-transformed for analysis, and then the results were back-transformed for reporting.

### Relationships among FABP3 and FABP4 with the other variables

The results of simple linear regression analysis showed that FABP3 was positively associated with age, duration of schizophrenia, hypertension, and creatinine (**Table 2**). In addition, sex, eGFR, hematocrit, hemoglobin, and HF HRV were negatively associated with levels of FABP3. Furthermore, FABP4 was positively associated with age, BMI, diabetes mellitus, hypertension, hyperlipidemia, SBP, LDL-C, triglycerides, creatinine, and FABP3. In addition, we found negative associations between eGFR, hematocrit, hemoglobin, and HF HRV with FABP4 level.

**Table 2:** Simple linear regression analysis between fatty acid-binding protein 3, fatty acid-binding protein 4 and other parameters.

	Log FABP 3		Log FABP 4	
Parameter	β	p-value	β	p-value
Age	0.248	<0.0001	0.131	0.033
Sex	-0.140	0.023	-0.113	0.067
Duration of schizophrenia	0.230	<0.0001	0.123	0.055
Body mass index	0.033	0.590	0.395	<0.0001
Diabetes Mellitus	0.099	0.107	0.310	<0.0001
Hypertension	0.154	0.012	0.199	0.001
Hyperlipidemia	0.104	0.091	0.123	0.045
Systolic blood pressure	0.086	0.164	0.168	0.006
Diastolic blood pressure	0.084	0.171	0.057	0.352
Total cholesterol	0.001	0.992	-0.010	0.867
High-density lipoprotein-cholesterol	0.055	0.381	-0.082	0.190
Low-density lipoprotein-cholesterol	-0.106	0.136	0.146	0.039
Triglycerides	-0.003	0.958	0.152	0.015
HbA1c	-0.095	0.223	0.102	0.188
Uric acid	-0.139	0.163	0.022	0.824
Albumin	0.072	0.520	0.141	0.202
Creatinine	0.383	<0.0001	0.323	<0.0001
Estimated glomerular filtration rate	-0.350	<0.0001	-0.334	<0.0001
Hematocrit	-0.260	<0.0001	-0.156	0.012
Hemoglobin	-0.244	<0.0001	-0.156	0.012
High sensitivity C-reactive protein^†^	-0.119	0.077	-0.104	0.120
White blood cell count	-0.036	0.559	0.007	0.916
Log FABP 3	–	–	0.476	<0.0001
HF HRV (ms^2^)^†^	-0.156	0.013	-0.163	0.009
LF HRV (ms^2^)^†^	-0.044	0.485	-0.021	0.739

FABP, fatty acid-binding protein; HF, absolute power of the high-frequency band; LF, absolute power of the low frequency band; HRV, heart rate variability. ^†^Significant difference was tested using log-transformed data.

After adjusting for age and sex, the results of multiple linear regression analysis showed a positive association between FABP3 and creatinine. In addition, levels of eGFR, hematocrit, hemoglobin, and HF HRV were negatively associated with levels of FABP3. Furthermore, a sex and age-adjusted multiple linear regression model revealed a positive association between FABP4 with BMI, diabetes mellitus, hypertension, SBP, LDL-C, triglycerides, creatinine, and FABP3. Moreover, we found negative associations between levels of HDL-C, eGFR, and HF HRV with FABP4 level (**Table 3**).

**Table 3:** Multiple linear regression analysis for fatty acid-binding protein 3, fatty acid-binding protein 4, serum biomarkers, and heart rate variability parameters.

	Log FABP 3		Log FABP 4	
Parameter	β*	p-value*	β*	p-value*
Body mass index	0.093	0.132	0.445	<0.0001
Diabetes mellitus	0.055	0.371	0.293	<0.0001
Hypertension	0.069	0.065	0.182	0.003
Hyperlipidemia	0.059	0.335	0.098	0.117
Systolic blood pressure	0.066	0.278	0.162	0.008
Diastolic blood pressure	0.047	0.443	0.080	0.198
Total cholesterol	-0.036	0.565	-0.040	0.534
High-density lipoprotein-cholesterol	-0.001	0.982	-0.150	0.026
Low-density lipoprotein-cholesterol	-0.119	0.094	0.157	0.027
Triglycerides	-0.007	0.914	0.149	0.017
HbA1c	-0.116	0.138	0.090	0.252
Uric acid	-0.086	0.412	0.049	0.645
Albumin	0.136	0.228	0.164	0.152
Creatinine	0.427	<0.0001	0.353	<0.0001
Estimated glomerular filtration rate	-0.413	<0.0001	-0.429	<0.0001
Hematocrit	-0.206	0.002	-0.117	0.079
Hemoglobin	-0.190	0.005	-0.117	0.087
High sensitivity C-reactive protein^†^	-0.073	0.262	-0.038	0.571
White blood cell count	-0.022	0.721	0.021	0.738
Log FABP 3	–	–	0.467	<0.0001
HF HRV (ms^2^)^†^	-0.151	0.014	-0.160	0.010
LF HRV (ms^2^)^†^	-0.042	0.490	-0.018	0.782

*Adjusted for age, sex by multiple linear regression analysis. HF, absolute power of the high-frequency band; LF, absolute power of the low frequency band; HRV, heart rate variability. ^†^Significant difference was tested using log-transformed data.

### HRV across tertiles of FABP3 and FABP4

In **Table 4**, the mean values and standard deviation of both LF and HF HRV are compared in the FABP3 and FABP4 tertiles. A significant and inverse association was found between FABP3 with HF HRV (p for trend = 0.008), and significant and inverse associations were found between FABP4 with both HF and LF HRV (p for trend = 0.007 and 0.017, respectively).

**Table 4:** Mean values and standard Deviation (SD) of heart rate variability across tertiles of fatty acid-binding protein 3 and fatty acid-binding protein 4.

	Tertiles of fatty acid-binding protein 3				
Parameter	First tertile ≤0.95 ng/mL	Second tertile 0.96-1.31 ng/mL	Third tertile >1.31 ng/mL	p for heterogeneity	p for trend
No.	88	88	89		
High frequency^†^	4.2±1.8	3.8±1.9	3.7±2.1	0.030	0.008
Low frequency^†^	4.4±1.8	4.2±1.7	3.9±1.9	0.187	0.070
	Tertiles of fatty acid-binding protein 4				
Parameter	First tertile ≤12 ng/mL	Second tertile 13-19.1 ng/mL	Third tertile >19.1 ng/mL	p for heterogeneity	p for trend
No.	89	88	88		
High frequency^†^	4.3±2.0	3.8±1.9	3.6±1.9	0.013	0.007
Low frequency^†^	4.4±1.9	4.3±1.6	3.8±1.8	0.049	0.017

^†^Significant difference was tested using log-transformed data.

## Discussion

In this study, we found a positive association between plasma FABP3 with creatinine, and negative associations between plasma FABP3 with eGFR, hematocrit, hemoglobin, and HF HRV after adjusting for sex and age. Moreover, we found positive associations between plasma FABP4 with BMI, diabetes mellitus, hypertension, SBP, LDL-C, triglycerides, creatinine, and FABP3, and negative associations between plasma FABP4 with HDL-C, eGFR, and HF HRV. Furthermore, a significant and inverse association was found between an increased concentration of plasma FABP3 with HF HRV (p for trend = 0.008), and significant and inverse associations were found between an increased concentration of plasma FABP4 with both HF and LF HRV (p for trend = 0.007 and 0.017, respectively). To the best of our knowledge, no previous investigation has reported relationships among FABP3 and FABP4 with HRV in patients with chronic schizophrenia. In the brain, FABP3 is expressed in neural stem/progenitor cells, neural progenitor cells and mature neurons (33). A previous study demonstrated that changes in the level of FABP3 in the brain may represent a disease mechanism in some patients with schizophrenia and autism spectrum disorder (34). Furthermore, as FABP3 binds and transports fatty acids, changes in FABP3 level could have an effect on various biological processes (35). Moreover, Maekawa et al. found that FABP4 knockout mice had changes in fatty acid composition in the cortex, and suggested that an ‘adipo-brain axis’ may underlie the pathophysiology of autism spectrum disorder, and that FABP4 could potentially be used as a biomarker (36). However, the biological mechanisms by which FABP3 and FABP4 are involved in the pathogenesis of low HRV are not well understood.

We previously found independent associations between a high level of FABP3 with plasma visfatin, high-sensitivity CRP, and white blood cell count, suggesting that the role of FABP3 in the pathophysiology of reduced ejection fraction in patients with stable angina may be through inflammatory processes (21). Furthermore, Hotamisligil et al. showed that FABP4 plays a role in many biological processes in addition to lipid metabolism regulation, including inflammation (37). FABP4 has been demonstrated to play a role in the pathogenesis of diseases mediated by inflammation, and FABP4 downregulation has been shown to reduce inflammation, oxidative stress and apoptosis (6, 38–40). In this study, we found that FABP3 and FABP4 were inversely associated with both HF and LF HRV (**Table 4**). Cooper et al. reported inverse associations between LF HRV with CRP, fibrinogen, and interleukin-6 (IL6), and inverse associations between HF HRV with CRP and fibrinogen, supporting the presence of a vagal anti-inflammation pathway (41). Furthermore, Alen et al. reported that HF HRV was strongly inversely associated with CRP, IL6 and fibrinogen, and that LF HRV was strongly inversely associated with IL6 and CRP, with or without adjustments for covariates. These findings are consistent with a cholinergic anti-inflammatory pathway, and suggest that parasympathetic modulation of inflammation through vagus nerves may affect certain inflammatory molecules more than others (42). In addition, Haensel et al. reported an inverse correlation between HRV with inflammatory markers in both healthy individuals and those with CVDs (16). These results may explain why high plasma FABP3 and FABP4 levels were inversely associated with HRV in our patients with chronic schizophrenia.

HRV can be used to noninvasively assess autonomic nervous system activity (43). A low HRV indicates a reduction in parasympathetic cardiac control, which has been linked to diabetes mellitus (44), sleep problems, and problems regulating emotional responses (45). Hence, HRV could be considered as a biomarker of general health and stress (46). FABP3 has been shown to be able to reliably diagnose AMI soon after symptom onset (47), and also to confirm or exclude the diagnoses of acute coronary syndromes. In addition, previous studies have shown that FABP3 may be useful to detect other conditions such as pulmonary thromboembolism, sepsis and congestive cardiac failure (48–50). In addition, increased serum levels of FABP3 have been reported in patients with metabolic syndrome and non-alcoholic fatty liver disease (51, 52), suggesting that FABP3 may serve as a biomarker of insulin resistance and subclinical myocardial damage. These findings highlight the role of FABP3 in regulating the autonomic nervous system with regards to cardiac and adipose tissue. Our results support (48–52) that high plasma FABP3 may be altered HRV through the autonomic nervous system regulation.

With regards to FABP4, evaluated levels of circulating FABP4 have been associated with arterial hypertension, type 2 diabetes mellitus, insulin resistance, obesity, atherosclerosis, cardiovascular disorders, abnormal QTc interval, kidney damage, cardiac dysfunction, and fatty liver disease (8, 22, 53–55). Our results showed significant and inverse associations between an increased concentration of plasma FABP4 with both HF and LF HRV (p for trend = 0.007 and 0.017, respectively); furthermore, in multiple linear regression model, we found that FABP4 was positively associated with BMI, diabetes mellitus, hypertension, SBP, LDL-C, triglycerides, creatinine, and FABP3. Moreover, we found negative associations between levels of eGFR, HDL-C and HF HRV with FABP4. Previous studies have reported increased concentrations of FABP4 in obese individuals, as well as positive correlations with insulin resistance, blood pressure, and waist circumference (56). Other studies have reported inverse associations between obesity and weight gain with changes in HRV (57–59). Various factors can influence HRV in obese individuals, including comorbidities, genetics, diet, emotional stress, and physical activity (60–63). Furthermore, type 2 diabetes mellitus has been associated with an overall decrease in HRV. Decreases in parasympathetic and sympathetic activity could be explained by changes in glucose metabolism having an adverse effect on HRV, resulting in cardiac autonomic neuropathy (64). Moreover, Liao et al. found that metabolic syndrome was associated with lower HRV (65). In addition, Chou et al. reported that the prevalence of autonomic dysfunction as assessed by HRV differed among patients with different stages of chronic kidney disease was different. Most patients with advanced chronic kidney disease have lower HRV (66). Drawz et al. also reported an association between lower HRV with a lack of exercise, heart failure, low eGFR, older age, and elevated phosphorus and HbA1c (67). Therefore, it is possible that FABP4 may be associated with obesity, chronic kidney disease, diabetes mellitus and metabolic syndrome, thereby contributing to lower HRV in patients with chronic schizophrenia.

## Limitations

There are several limitations in our study. First, concerning the effect of existence of peripheral neuropathy in diabetics that would reduce HRV, as our study patients with chronic schizophrenia may be unable to communicate well during the sensory nerve function examination, we did not determine the relationship between the diabetic sensory and motor neuropathy with HRV. Although diabetics peripheral neuropathy of sensory nerve always noted in older diabetic patients and might relative the oscillation changes of heart rate (68). The median age of the patients were 56 years (interquartile range, 48-64 years) in the present study, and previous study showed the change of autonomic function is earlier than the impaired of diabetic sensory and motor function (68). Hence, we believe the diabetics peripheral neuropathy might not be significant confounder to affect the HRV data of present study. Second, we did not compare our results with healthy population is because the antipsychotic or psychotherapy theoretically will influence HRV measurement. It is hard to eliminate these interventional effects while compared the HRV result of patient with schizophrenia to with normal population. However, this limit the applicability of our results to normal healthy population and other groups. Third, HRV is also strongly influenced by stress and emotional change. To eliminate the influence of the schizophrenia symptom to our result as possible as we can, all patients in this study are selected from the chronic psychiatric ward with relative stable status of schizophrenia, they are in relative calm condition with no significant negative or positive symptom, and more willing to receive the HRV examination. We believe this population is a good candidate to investigate the risk factor associated with cardiovascular disease in patients with schizophrenia. Fourth, the posture and breathing of examination also influences the HRV result. Although there are several protocols available in HRV measurements, in the present study, HRV were measured by using the short-term assessment of HRV. We performed the test at resting supine position with normal spontaneous breathing in this study. Because this is the only way, we could ensure all of our patients performed the test in same condition. Previous study showed that short-term HRV measurement was more reliable at rest than at tilt or pharmacological stimulation (69). In the other hand, HRV showed overall more reliable during spontaneous breathing especially in chronic obstructive pulmonary disease population (70), which similar with patients with schizophrenia, easy influenced by stress and emotional change. Other limitations of this study also include the cross-sectional design, which hinders the ability to examine causal relationships between increased plasma FABP3/FABP4 levels with lower HRV. Long-term follow-up studies are warranted to investigate the association of FABP3 and FABP4 with lower HRV further. In addition, the study cohort was relatively small. Cross-sectional studies are useful in determining prevalence, however they do not permit robust comparisons. Further studies with larger multi-ethnic cohorts should be conducted to explore the associations found in this study. Finally, further studies are needed to assess whether elevated FABP3 and FABP4 levels are associated with lower HRV.

In summary, we found an inverse association between FABP3 with HF HRV, and inverse associations between FABP4 with both LF and HF HRV in patients with chronic schizophrenia. Increased FABP3 and FABP4 levels may be associated with CVD-related health problems in patients with chronic schizophrenia. Our results may imply that regular monitoring of HRV is warranted in patients with chronic schizophrenia to identify the distribution of the major heart rate oscillations (the LF and HF bands), and that reduced HRV is associated with an increased risk of CVD morbidity and mortality. The benefits of improving low HRV in patients with chronic schizophrenia may also extend to improving cardiovascular health.

## Data availability statement

The original contributions presented in the study are included in the article/**Supplementary material**. Further inquiries can be directed to the corresponding author.

## Ethics statement

The studies involving human participants were reviewed and approved by The Human Research Ethics Committee of Kaohsiung E-Da Hospital. The patients/participants provided their written informed consent to participate in this study.

## Author contributions

All authors contributed to this study. W-CH and W-HT conceived and designed the study. W-CH, Y-JL, and W-HT provided the methodology. F-MC performed the formal analysis, and project administration. W-CH and W-HT validated the data. T-HY, C-CW, T-LL, I-TT, C-FH, C-YC, and W-HT performed the investigation, resources, and data curation. T-HY, C-CW, T-LL, I-TT, C-FH, C-YC, and W-HT prepared the manuscript. W-CH, T-HY, C-CW, T-LL, I-TT, C-FH, C-YC, Y-JL, and W-HT reviewed and edited the manuscript. W-CH, Y-JL, and W-HT performed the visualization. W-HT performed the supervision and funding acquisition. All authors contributed to the article and approved the submitted version.
